# NMN reverses D-galactose-induced neurodegeneration and enhances the intestinal barrier of mice by activating the Sirt1 pathway

**DOI:** 10.3389/fphar.2025.1545585

**Published:** 2025-04-10

**Authors:** Yuxian Lin, Yajing Wang, Xinxin Yang, Ziwei Ding, Mingye Hu, Xianfeng Huang, Qichun Zhang, Yingcong Yu

**Affiliations:** ^1^ The Third Clinical Institute Affiliated to Wenzhou Medical University, Wenzhou, Zhejiang, China; ^2^ School of Pharmacy, Yantai University, Yantai, Shandong, China; ^3^ School of Pharmacy, Changzhou University, Changzhou, Jiangsu, China; ^4^ Department of Infectious Diseases, Wenzhou Third Clinical Institute Affiliated to Wenzhou Medical University, The Third Affiliated Hospital of Shanghai University, Wenzhou People’s Hospital, Wenzhou, China; ^5^ School of Pharmacy, Nanjing University of Chinese Medicine, Nanjing, Jiangsu, China

**Keywords:** aging, NMN, oxidative stress, neuroinflammation, intestinal barrier, SIRT1

## Abstract

**Background:**

Age-related decline in nicotinamide adenine dinucleotide (NAD+)—a central regulator of cellular metabolism, DNA repair, and immune homeostasis—is strongly associated with physiological dysfunction. Nicotinamide mononucleotide (NMN), a potent NAD+ precursor, shows promise in counteracting aging-related pathologies, particularly neurodegenerative decline.

**Methods:**

An aging model was established in mice through 8-week D-galactose (D-gal) exposure, followed by NMN oral supplementation. Behavioral outcomes (open field test, Morris water maze) were analyzed alongside oxidative stress markers (SOD, CAT, AGEs), inflammatory cytokines (TNF-α, IL-1β, IL-6, IL-10), and neurotransmitters (LC-MS/MS). Apoptotic activity (TUNEL, p16/p21), mitochondrial regulators (Sirt1, p-AMPK, PGC-1α), and intestinal barrier integrity (HE/AB-PAS staining) were evaluated. Sirt1 dependency was confirmed using inhibitor Ex527.

**Results:**

NMN restored locomotor activity and spatial memory in D-gal mice without altering body weight. Mechanistically, NMN synergistically attenuated oxidative stress and systemic inflammation, elevating antioxidant enzymes (SOD, CAT) and IL-10 while suppressing pro-inflammatory cytokines (TNF-α, IL-6) and AGEs. Cortical/hippocampal analyses revealed reduced apoptosis (TUNEL^+^ cells) and senescence markers (p16, p21), with enhanced mitochondrial function via Sirt1/AMPK/PGC-1α activation (Sirt1, p-AMPK). NMN concurrently preserved intestinal mucosal architecture, mitigating D-gal-induced barrier disruption. Crucially, all benefits were abolished by Sirt1 inhibition, confirming pathway specificity.

**Conclusion:**

Our findings establish NMN as a multifaceted therapeutic agent that preserves neurocognitive function and intestinal homeostasis in aging models by orchestrating antioxidative, anti-inflammatory, and antiapoptotic responses through Sirt1/AMPK/PGC-1α activation. This work provides translational insights into NAD+-boosting strategies for age-related disorders.

## Graphical Abstract

**Figure:**
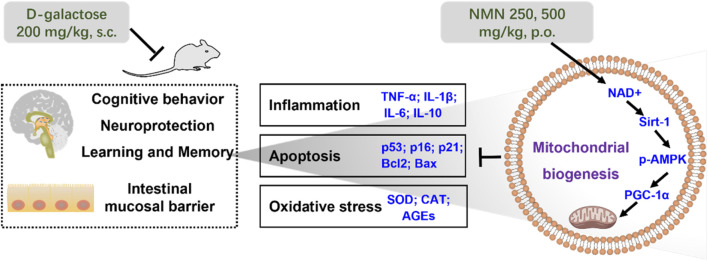

## Introduction

The global population aged 60 years and older is projected to double from 800 million in 2015 to 2 billion by 2050, emphasizing the urgent need for effective therapeutic strategies to mitigate age-related chronic diseases. While advancements in medicine have extended human life expectancy, they have also led to a rising prevalence of conditions such as Alzheimer’s disease (AD), cardiovascular disease, and cancer-diseases that, despite their diverse clinical presentations, share common pathological features, including cellular senescence, mitochondrial dysfunction, and chronic inflammation (Farrelly and Daly, 2024; Ledford, 2024).

Non-resolving chronic inflammation, a hallmark of aging, has been identified as a major driver of pathological aging processes. It contributes to the dysfunction of hematopoietic stem cells, accelerates senescence, and perpetuates neuroinflammation, all of which are implicated in cognitive decline and neurodegeneration, particularly in Alzheimer’s disease (Heneka et al., 2024; Riessland et al., 2024). Chronic neuroinflammation exacerbates tau protein hyperphosphorylation, disrupts mitochondrial function, and induces oxidative stress (OS), all of which accelerate neurodegeneration. Signaling pathways such as Sirt1 and cGAS/STING have been identified as central regulators of aging-associated inflammation and neurodegeneration, presenting attractive therapeutic targets (Kalinkovich and Livshits, 2024). Mitochondrial dysfunction further exacerbates chronic inflammation through the aberrant accumulation of metabolic intermediates, such as succinate and itaconate, derived from the tricarboxylic acid (TCA) cycle (Kong et al., 2024). Excess succinate, for instance, stabilizes hypoxia-inducible factor-1α (HIF-1α), inhibits prolyl hydroxylase, and promotes interleukin-1β (IL-1β) production, sustaining inflammatory signaling (Salminen et al., 2015). These mechanisms underscore the critical role of mitochondria not only as cellular energy providers but also as regulators of inflammation and aging.

The human brain, consisting of approximately 85 billion neurons, undergoes structural and functional changes with aging that culminate in cognitive decline and memory impairment. Neurons are highly reliant on mitochondrial ATP production; thus, mitochondrial dysfunction and the subsequent increase in ROS generation can significantly contribute to neurodegenerative processes (Sies and Jones, 2020). Age-related mitochondrial dysfunction involves the accumulation of mutations in mitochondrial DNA (mtDNA), decreased oxidative phosphorylation efficiency, and impaired antioxidant defenses, leading to oxidative damage and cellular aging (Byrns et al., 2024).

The sirtuin family, particularly Sirt1, has emerged as a critical regulator of aging and longevity due to its role as a NAD^+^-dependent deacetylase. Sirt1 governs various cellular processes, including DNA repair, mitochondrial biogenesis, autophagy, and inflammation, primarily through the activation of downstream pathways such as AMPK and PGC-1α (Packer, 2020; Satoh et al., 2017). By modulating key proteins like p53, NF-κB, and the forkhead box O (FOXO) transcription factors, Sirt1 plays a pivotal role in promoting cellular resilience and neuroprotection (Fujita and Yamashita, 2018). Activation of the Sirt1/AMPK/PGC-1α axis has been shown to improve mitochondrial function, mitigate apoptosis, and enhance autophagy in neurodegenerative disease models, highlighting its therapeutic potential.

NAD^+^, a vital coenzyme in cellular metabolism, is central to mitochondrial function, DNA repair, and the regulation of cellular aging. Age-related depletion of NAD^+^ impairs these processes, exacerbating the progression of neurodegenerative diseases and other age-associated pathologies (Covarrubias et al., 2021). Supplementation with NAD^+^ precursors, such as nicotinamide mononucleotide (NMN), has emerged as a promising strategy to restore NAD^+^ levels, thereby improving mitochondrial bioenergetics, activating longevity pathways like Sirt1, and mitigating chronic inflammation (Rajman et al., 2018). Recent studies have demonstrated the efficacy of NMN and other NAD^+^ precursors in enhancing health span and reversing age-related functional decline (Wu et al., 2023).

The gastrointestinal (GI) tract, a critical interface for systemic health, harbors the body’s largest population of immune cells and is a key component of the brain-gut axis. Aging compromises intestinal barrier integrity and increases gut permeability, allowing endotoxins to enter the systemic circulation and contribute to chronic inflammation and neuroinflammation (Connell et al., 2022). Disruption of the gut barrier may exacerbate systemic inflammatory responses, further implicating the brain-gut axis in aging-associated neurodegeneration. Recent studies suggest that NAD^+^ precursors, such as NMN, may ameliorate intestinal barrier dysfunction, highlighting a potential avenue for modulating age-related systemic and neuroinflammatory processes (Li et al., 2024).

In the present study, we utilized a D-galactose (D-gal)-induced aging mouse model to investigate the potential of NMN in reversing age-related cognitive impairment, neuroinflammation, and intestinal barrier dysfunction. NMN supplementation significantly improved cognitive performance, reduced systemic and neuroinflammatory markers, and enhanced mitochondrial function while ameliorating oxidative stress. Importantly, the co-administration of Ex-527, a pharmacological Sirt1 inhibitor, attenuated these beneficial effects, confirming that NMN exerts its protective actions via activation of the Sirt1/AMPK/PGC-1α pathway.

Our findings provide compelling evidence that NMN supplementation mitigates age-related neurodegenerative symptoms and systemic dysfunction by restoring NAD^+^ homeostasis and activating Sirt1-dependent pathways. This study highlights the therapeutic potential of NMN as a promising intervention to counteract cellular and molecular aging, offering translational relevance for combating age-associated neurodegenerative and inflammatory diseases.

## Materials and methods

### Animal model

In this study, we employed a male-specific pathogen-free (SPF) grade C57BL/6 murine model to evaluate the therapeutic effects of nicotinamide mononucleotide (NMN) on aging phenotypes induced by D-gal. A total of 60 male mice (6–8 weeks old, weighing 20 ± 1 g) were obtained from Hangzhou Medical College and acclimatized for 1 week under standardized conditions (temperature: 25°C ± 1°C; relative humidity: 60% ± 5%) with free access to food and water. Following acclimatization, animals were weighed, identified, and randomly assigned to five experimental groups: Group 1 (Normal), Group 2 (D-gal model), Group 3 (D-gal + NMN 250 mg/kg), Group 4 (D-gal + NMN 500 mg/kg), and Group 5 (D-gal + NMN 500 mg/kg + Ex527 5 mg/kg). The oral dosage of NMN was designed as previous literature (Yoshino et al., 2018). The aging model was induced by subcutaneous administration of D-gal (200 mg/kg/day) in all groups except the Control group. Groups 3 and 4 received NMN (purity 99.9%, Yuyao Lifespan Health Technology Co., Ltd., Zhejiang, China) by oral gavage, while Group 5 was co-administered NMN (500 mg/kg) and Ex527, a selective Sirt1 inhibitor, administered intraperitoneally at 5 mg/kg. The intervention period spanned 8 weeks, during which body weight was monitored longitudinally. All experimental procedures were conducted in strict compliance with the Guide for the Care and Use of Laboratory Animals (NIH Publication No. 80-23, revised 1978). Ethical approval for the study was obtained from the Animal Care and Use Committee of the Nanjing University of Chinese Medicine (Approval No. 202302A085).

### Open-field test (OFT)

Following the 8-week treatment period, the open-field test (OFT) was conducted to evaluate locomotor activity and anxiety-like behavior in aging mice, as previously described (Ge et al., 2023). The OFT is a well-established behavioral assay that provides quantitative measurements of exploratory behavior, general locomotion, and anxiety-like responses in rodents. The test was performed using a mouse locomotor activity monitoring system (50 cm × 50 cm×40 cm, JLBehv-LAM-4; Jiliang Ltd., Shanghai, China) under standardized conditions. Prior to testing, the experimental chamber was thoroughly cleaned and disinfected to eliminate residual odors, particularly fecal or urinary traces, which could influence behavioral outcomes. Mice were acclimated to the testing room for at least 1 h before the experiment to minimize environmental stressors. Each mouse was carefully removed from its home cage with minimal handling stress, ensuring the animal’s back was facing the experimenter during transfer, and gently placed in the center of the open field arena. Behavioral recording commenced immediately, capturing parameters such as total distance traveled and average velocity over a 5-minute observation period. To control for circadian influences on activity, all testing was conducted within a 24-hour time window under consistent lighting conditions. Post-assessment, mice were promptly returned to their home cages. The testing apparatus was disinfected with 70% ethanol and wiped clean between trials to prevent residual olfactory cues from confounding subsequent recordings. A quiet and undisturbed environment was maintained throughout the procedure to minimize external disturbances that could impact the behavioral outcomes.

### Morris water maze (MWM)

Following the 8-week treatment regimen, spatial learning and memory performance in the mice was assessed using the Morris water maze (MWM) test, as previously described (Zhao et al., 2023). The MWM is a widely validated behavioral assay for evaluating hippocampus-dependent spatial navigation and memory in rodents. The specifications of the Morris water maze for mice are as follows: circular pool diameter 120 cm, 50 cm height, platform diameter 8 cm in the fourth quadrant, and submersion level of 1.5 cm.

The MWM procedure consisted of a 5-day acquisition phase, followed by a probe trial on day 6. During the acquisition phase, mice underwent four training trials per day to locate a submerged escape platform hidden in a circular pool filled with water maintained at 20°C–22°C. At the start of each trial, mice were released from a randomized starting point, and the escape latency-defined as the time taken to reach the hidden platform-was recorded, with a maximum trial duration of 60 s. Mice that failed to locate the platform within the allotted time were gently guided to the platform and allowed to remain there for 60 s to facilitate spatial learning; their escape latency was recorded as 120 s. On the sixth day, a probe trial was conducted to assess memory retention in the absence of the escape platform. Mice were released into the pool and allowed to swim freely for 60 s. The parameters measured included the frequency of crossings over the target platform location, the time spent in the target quadrant, and the total distance traveled within the target quadrant. These parameters were quantified using an automated animal behavior analysis system (Zhenghua Ltd., Shanghai, China). To minimize variability, all experiments were conducted at consistent times of the day to control for diurnal influences on behavior. The water temperature was strictly maintained to reduce thermal stress and ensure consistency across trials. Metrics such as escape latency, path length, and swimming velocity were meticulously recorded to provide a robust evaluation of spatial learning and memory.

### Determination of superoxide dismutase (SOD) and catalase (CAT)

Mice were anesthetized with 1% sodium pentobarbital at a dose of 50 mg/kg, and blood samples were collected from the orbital venous plexus using sterile capillary tubes. The collected blood was allowed to clot overnight at 4°C and subsequently centrifuged at 3,000 rpm for 10 min to isolate the serum. SOD and CAT activities were quantified using the nitroblue tetrazolium photoreduction assay and the guaiacol oxidation method, respectively, in accordance with the manufacturer’s protocols (Huaying Ltd., Beijing, China).

### Cytokines analysis

Mice were euthanized via cervical dislocation, and brain tissues were promptly harvested on ice to maintain tissue integrity and biochemical stability. The tissues were rinsed with ice-cold (4°C) saline to remove residual blood, and excess moisture was gently blotted. Brain tissues were weighed for accurate normalization, followed by homogenization in ice-cold saline at a mass-to-volume ratio of 1:9 (g/mL) using a mechanical homogenizer in an ice-water bath. The resulting homogenates were centrifuged at 3,500 rpm for 10 min at 4°C to remove cellular debris. The supernatant was carefully collected and stored at −80°C for subsequent biochemical analyses. Quantification of AGEs, tumor necrosis factor-alpha (TNF-α), interleukin-1 beta (IL-1β), interleukin-6 (IL-6), and interleukin-10 (IL-10) was performed using validated radioimmunoassay kits (Huaying Ltd., Beijing, China), in accordance with the manufacturer’s protocols. These analytes serve as critical biomarkers for evaluating neuroinflammatory and neurodegenerative processes. All procedures were conducted under rigorous quality control to ensure assay reliability and reproducibility.

### Neurotransmitter analysis

Following euthanasia via cervical dislocation, brain tissues were rapidly excised and placed on ice to preserve neurotransmitter integrity, as previously described (Ge et al., 2023). The tissues were accurately weighed and homogenized in a tenfold volume of ice-cold 0.1% formic acid solution to facilitate cellular disruption and neurotransmitter release. Homogenization was performed for 3 min under controlled conditions to ensure complete cell lysis. An aliquot of 100 μL of the homogenate was subsequently mixed with 400 μL of ice-cold 0.2% formic acid-acetonitrile solution, vortexed for 3 min to precipitate proteins and enhance neurotransmitter extraction. The mixture underwent centrifugation at 12,000 rpm for 10 min at 4°C, enabling separation of the supernatant containing the target analytes from cellular debris and insoluble components. The resulting supernatant was carefully transferred to a centrifugal concentrator and dried under vacuum to remove organic solvents, facilitating sample concentration. The dried residues were reconstituted in 0.1% formic acid to ensure solubility and optimal ionization for subsequent mass spectrometric analysis. The reconstituted samples were centrifuged at 12,000 rpm for 10 min at 4°C to remove residual particulates, and the resulting clear supernatants were filtered to ensure compatibility with the mass spectrometry system. Quantitative analysis of neurotransmitters was performed using an AB SCIEX Triple Quad™ 6500 triple quadrupole linear ion trap mass spectrometer. Targeted ion pairs and mass spectrometry parameters for neurotransmitter detection are summarized in Table 1. All procedures were conducted under stringent quality control to ensure analytical precision and reproducibility.

**TABLE 1:** Ion pairs for neurotransmitters in MS.

Indicator	Q1→Q3/(m/z)	DP/V	CE /V
γ- aminobutyric acid (GABA)	104.0→87.2	36	13
Glutamate (Glu)	148.0→84.3	105	19
Dopamine (DA)	154.1→137.1	130	13
5-hydroxytryptamine(5-HT)	177.1→160.1	130	17
Epinephrine(E)	184.4→166.2	21	10
Norepinephrine (NE)	170.1→107.2	40	31

### Western blotting

Following euthanasia via cervical dislocation, brain tissues were rapidly harvested on ice to preserve protein integrity. Tissues were homogenized in radioimmunoprecipitation assay (RIPA) buffer supplemented with protease and phosphatase inhibitor cocktails to prevent protein degradation and dephosphorylation. The homogenates were centrifuged at 4°C to isolate the soluble protein fraction, and protein concentrations were determined using the bicinchoninic acid (BCA) assay, ensuring precise quantification of total protein content. For electrophoretic separation, protein samples were denatured under reducing conditions with dithiothreitol (DTT) and sodium dodecyl sulfate (SDS), ensuring uniform negative charges and size-dependent migration. Samples were resolved via SDS-PAGE on polyacrylamide gels. An initial voltage of 70 V was applied for 20 min to concentrate proteins within the stacking gel, followed by 110 V for 60 min to achieve separation in the resolving gel. Following electrophoresis, proteins were transferred onto polyvinylidene fluoride (PVDF) membranes using a wet-transfer system, ensuring efficient immobilization. Membranes were blocked with 5% non-fat milk in Tris-buffered saline containing 0.1% Tween-20 (TBST) to minimize nonspecific binding. Primary antibodies specific to SOD1 (1:10000), SOD2 (1:2000), Bax (1:5000), Bcl-2 (1:2000), Sirt1 (1:1000), p16 (1:5000), p21 (1:1000), p-AMPK (1:5000), AMPK (1:2000), PGC-1α (1:1000), and GAPDH (1:10000) were incubated overnight at 4°C to optimize antigen-antibody interactions. Antibodies against SOD1, SOD2, Bax, Bcl-2, Sirt1, p16, p21, p-AMPK, and AMPK were purchased from Abcam (United States); PGC-1α was sourced from Absin (China), and GAPDH from Cell Signaling Technology (CST, United States). After three washes in TBST to remove unbound primary antibodies, membranes were incubated for 2 h at room temperature with horseradish peroxidase (HRP)-conjugated secondary antibodies (CST, United States). Excess secondary antibodies were removed through additional TBST washes. Immunoreactive protein bands were visualized using enhanced chemiluminescence (ECL) substrates, and band intensities were quantified using ImageJ 22 software (National Institutes of Health, United States) to provide precise, semi-quantitative analysis of protein expression levels.

### Terminal deoxynucleotidyl transferase dUTP nick-end labelling assay

Mice were anesthetized with an intraperitoneal injection of sodium pentobarbital (50 mg/kg). Following anesthesia, the chest cavity was carefully opened to expose the heart. The right atrium was incised, and a perfusion needle was inserted into the left ventricle. Initial perfusion was performed with saline to flush out blood, continuing until the effluent from the right atrium became clear. Subsequently, the mice were perfused with 4% paraformaldehyde for tissue fixation, with the process continuing until the liver tissue appeared white. The brain was then harvested and placed in 4% paraformaldehyde for a minimum of 24 h for post-fixation. Following fixation, the tissues underwent dehydration, and were subsequently embedded in paraffin, a standard procedure that preserves tissue morphology for further histological analysis. Paraffin-embedded tissues were sectioned into 5-μm-thick slices for further examination. To assess apoptosis, the tissue sections were subjected to the terminal deoxynucleotidyl transferase dUTP nick-end labeling (TUNEL) assay, a widely used and highly sensitive method for detecting DNA fragmentation, a characteristic feature of apoptotic cell death. The TUNEL assay was conducted using a commercially available TUNEL kit (Saver, Wuhan, China), following the manufacturer’s protocol. The TUNEL-positive cells, indicative of DNA fragmentation and apoptotic cell death, were visualized under a fluorescence microscope. Quantification of apoptotic cells was performed by counting the number of TUNEL-positive cells within the brain tissue sections, providing an assessment of apoptosis in the experimental model.

### Intestinal H&E staining and Alcian Blue (AB)-Periodic Acid-Schiff (PAS) staining

Colonic tissues were harvested from mice fixed with 4% paraformaldehyde via left ventricle perfusion as described above. Following fixation, the tissues underwent a dehydration protocol and were subsequently embedded in paraffin. Thin sections (5 μm) were cut for histological analysis. For general tissue morphology, sections were stained with hematoxylin and eosin (H&E) using a standard procedure, allowing for detailed examination of the intestinal architecture. For Alcian Blue (AB)-Periodic Acid-Schiff (PAS) staining, sections were first incubated with Alcian blue (pH 2.5) for 30 min to visualize acidic mucopolysaccharides, which prominently stain goblet cells responsible for mucus secretion. After washing, PAS staining was performed to detect neutral mucopolysaccharides and eosinophils. This was achieved through periodic acid oxidation followed by Schiff’s reagent application, and counterstaining with hematoxylin for nuclear visualization. This dual staining technique facilitates a comprehensive evaluation of the intestinal barrier, offering critical insights into its structural and functional integrity, which are pivotal for studies on gastrointestinal physiology and pathology.

### Statistical analysis

Statistical analysis was performed using GraphPad Prism version 8.0.1. Data are presented as the mean ± standard error of the mean (SEM). To evaluate the significance of differences between groups, one-way analysis of variance (ANOVA) was conducted. Post-hoc analysis was carried out using Tukey’s Honestly Significant Difference (HSD) test for multiple comparisons. Statistical significance was defined at a threshold of p < 0.05.

## Results

### Temporal Analysis of body weight

**FIGURE 1:**
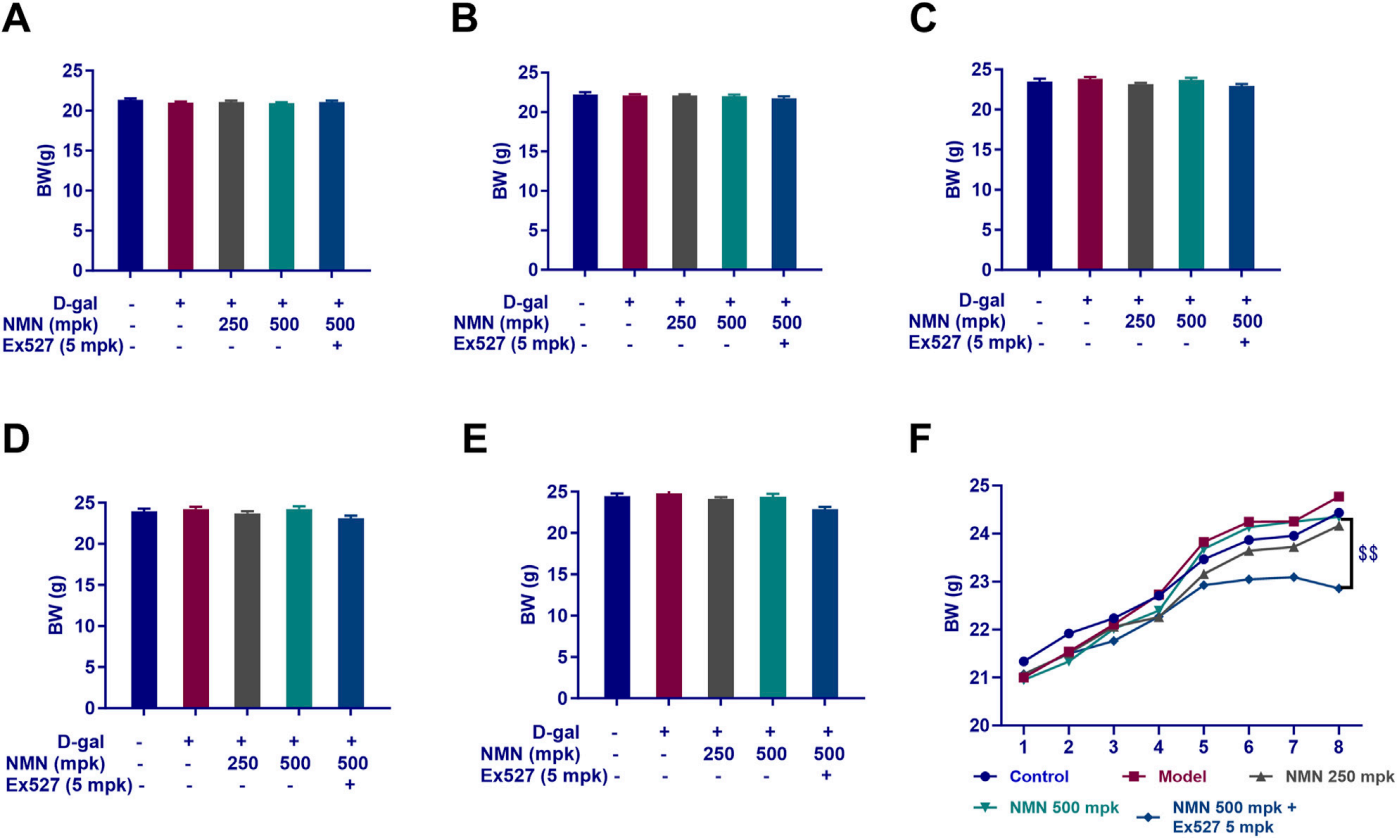
Temporal Analysis of Murine Body Weight. **(A–E)** The body weight measurements at distinct time points: Week 1 **(A)**, Week 3 **(B)**, Week 5 **(C)**, Week 7 **(D)**, and Week 8 **(E)**, following the corresponding treatment regimens; **(F)** A comprehensive summary of the body weight trajectory over the 8-week experimental period. Each data point represents the mean ± SEM, with n = 12; $$, p < 0.01, in comparison to the NMN 500 mg/kg group; mpk, mg/kg.

Body weight measurements were conducted at weeks 1, 3, 5, 7, and 8, with the results presented in Figures 1A–E. The trajectory of body weight changes over the 8-week experimental period is shown in Figure 1F. Analysis of the weight curves revealed no statistically significant differences between groups during the first 4 weeks of the study (Figures 1B, F). However, a divergence in weight gain became evident from week 5 (Figures 1C, F). Notably, in the group receiving NMN at a dose of 500 mg/kg in combination with Ex527 (5 mg/kg), a significant reduction in mean body weight was observed by week 8 (p < 0.01), indicating a marked inhibitory effect of Ex527 on body weight gain in mice.

### NMN ameliorates locomotor activity and enhances learning and memory in aging mice

**FIGURE 2:**
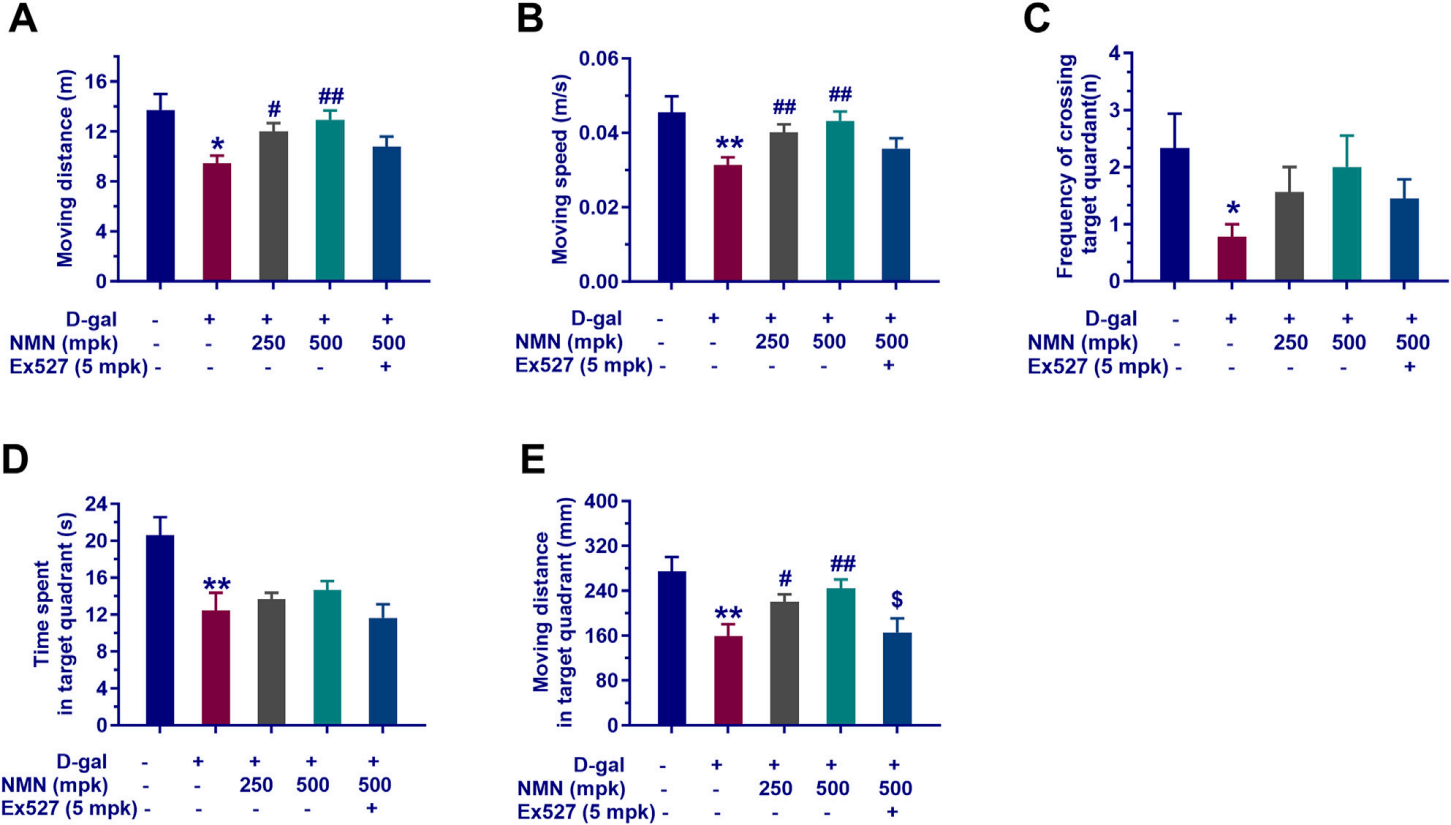
NMN ameliorates locomotor activity and enhances learning and memory in aging mice. **(A, B)** The locomotor moving distance and the velocity assessed over a 5-min period in the OFT; **(C–E)** The frequency of target quadrant crossings, residence time, and moving distance within the target quadrant of MWM, respectively. Each data point represents the mean ± SEM, with n = 9; *, p < 0.05, **, p < 0.01, compared to the Normal group; #, p < 0.05, ##, p < 0.01, versus the D-gal group; $, p < 0.05, in comparison to the NMN 500 mpk group.

To assess the effects of NMN on locomotor activity, as well as spatial learning and memory in aging mice, a comprehensive behavioral evaluation was performed using the OFT and MWM. As shown in the OFT, administration of NMN at doses of 250 mg/kg and 500 mg/kg resulted in a significant increase in locomotor activity, as evidenced by a greater total distance traveled (Figure 2A; p < 0.05 and p < 0.01) and higher moving speed (Figure 2B; p < 0.01) in aging mice, relative to the model group. These findings suggest that NMN enhances both motor function and locomotor activity in aged mice. In the MWM test, D-galactose-induced aging mice exhibited a significantly reduced number of target quadrant crossings (p < 0.05), shorter time spent in the target quadrant (p < 0.01), and decreased distance traveled within the target quadrant (p < 0.01), compared to the control group, indicating impaired spatial learning and memory. Administration of NMN at 250 mg/kg and 500 mg/kg increased the number of target quadrant crossings (Figure 2C) and residence time in the target quadrant (Figure 2D), suggesting an improvement in spatial memory, although these differences did not reach statistical significance. However, a significant increase in the total distance traveled (p < 0.01) in both the NMN 250 mg/kg and NMN 500 mg/kg groups was observed, indicating enhanced spatial learning (Figure 2E). Co-administration of Ex527, an inhibitor of Sirt1, appeared to attenuate the effects of NMN, although the difference did not achieve statistical significance. This suggests a potential interaction between NMN and Sirt1 signaling pathways in the regulation of aging and cognitive function. Collectively, these results indicate that NMN effectively mitigates age-related cognitive decline in D-gal-induced aging mice. The modulation of NMN’s effects by Ex527 highlights the complexity of these interactions, warranting further investigation into the underlying mechanisms.

### NMN reduces the levels of OS in aging mice

**FIGURE 3:**
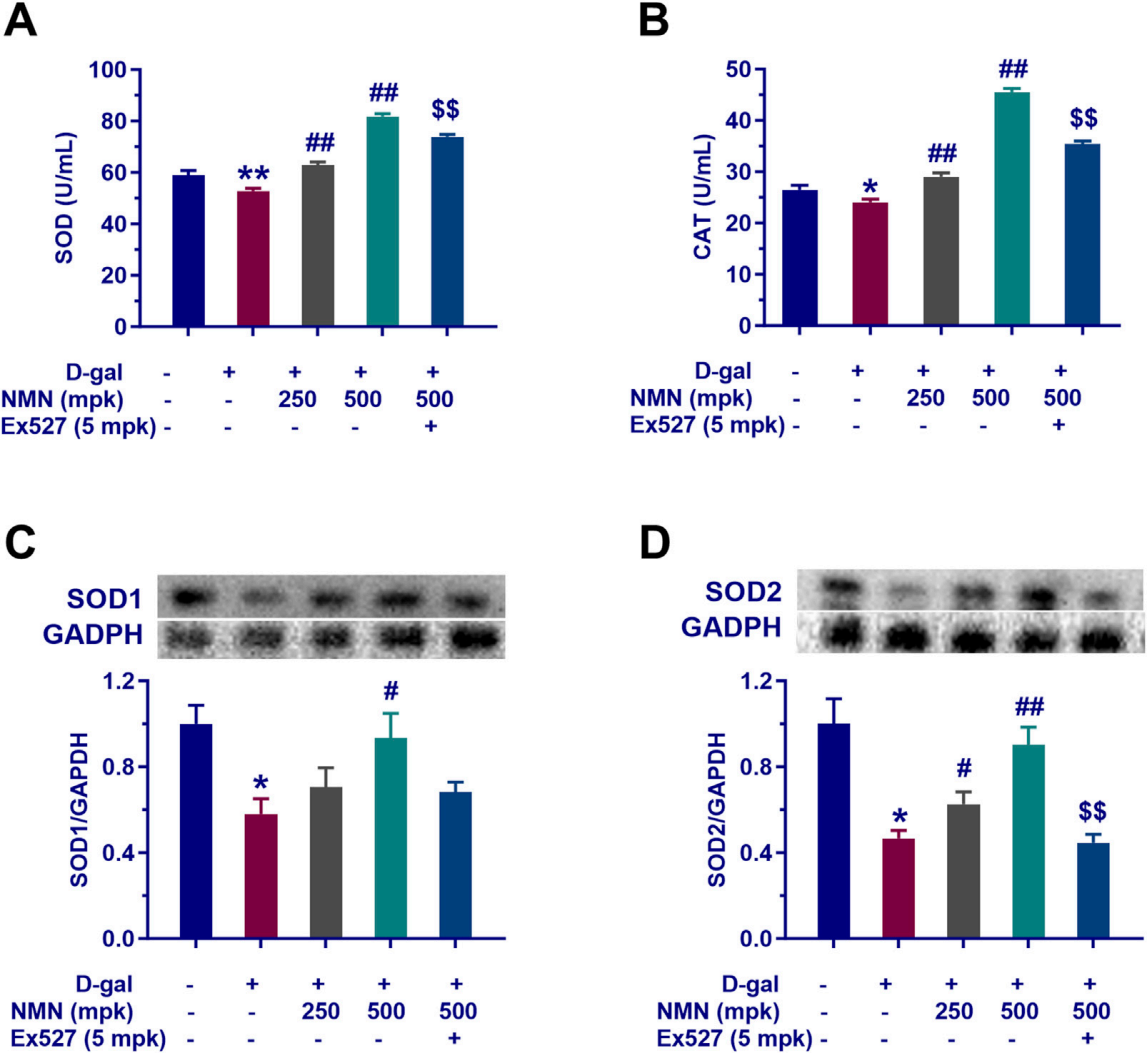
NMN attenuates oxidative stress of aging mice. **(A, B)** The activities of SOD and CAT in serum of mice determined via biochemical assay following the corresponding treatment regimens; **(C, D)** The expression levels of SOD1 and SOD2 in the brain of mice analysed by Western blotting. Each data point represents the mean ± SEM, with n = 12; *, p < 0.05, **, p < 0.01, compared to the Normal group; #, p < 0.05, ##, p < 0.01, versus the D-gal group; $$, p < 0.01, in comparison to the NMN 500 mpk group.

NMN has been shown to mitigate oxidative stress (OS) in aging mice, a key factor in the progression of aging and various degenerative and chronic conditions, including inflammation, cancer, arthritis, neurodegenerative diseases, and cardiovascular disorders. To quantitatively assess the impact of NMN on OS, we measured the expression levels of SOD and CAT in serum, and SOD1 and SOD2 in brain tissue from aging mice. Our results demonstrated a significant reduction in all these OS biomarkers following 8 weeks of D-gal administration, consistent with the accumulation of reactive oxygen species (ROS) commonly observed in aging models. Notably, administration of NMN at doses of 250 mg/kg and 500 mg/kg significantly increased serum SOD and CAT levels (p < 0.01, Figures 3A, B), and brain SOD1 and SOD2 expression (p < 0.01, Figures 3C, D), indicating a substantial enhancement in endogenous antioxidant defense mechanisms. These effects were notably attenuated by co-administration of Ex527, a Sirt1 inhibitor (p < 0.01), suggesting a potential role of the Sirt1 pathway in mediating the antioxidant effects of NMN. In summary, these findings suggest that NMN can effectively restore the balance between ROS and antioxidants, thereby alleviating the detrimental effects of oxidative stress in both serum and brain tissues of D-gal-induced aging mice.

### NMN alleviates the inflammatory symptoms and reduces AGEs

**FIGURE 4:**
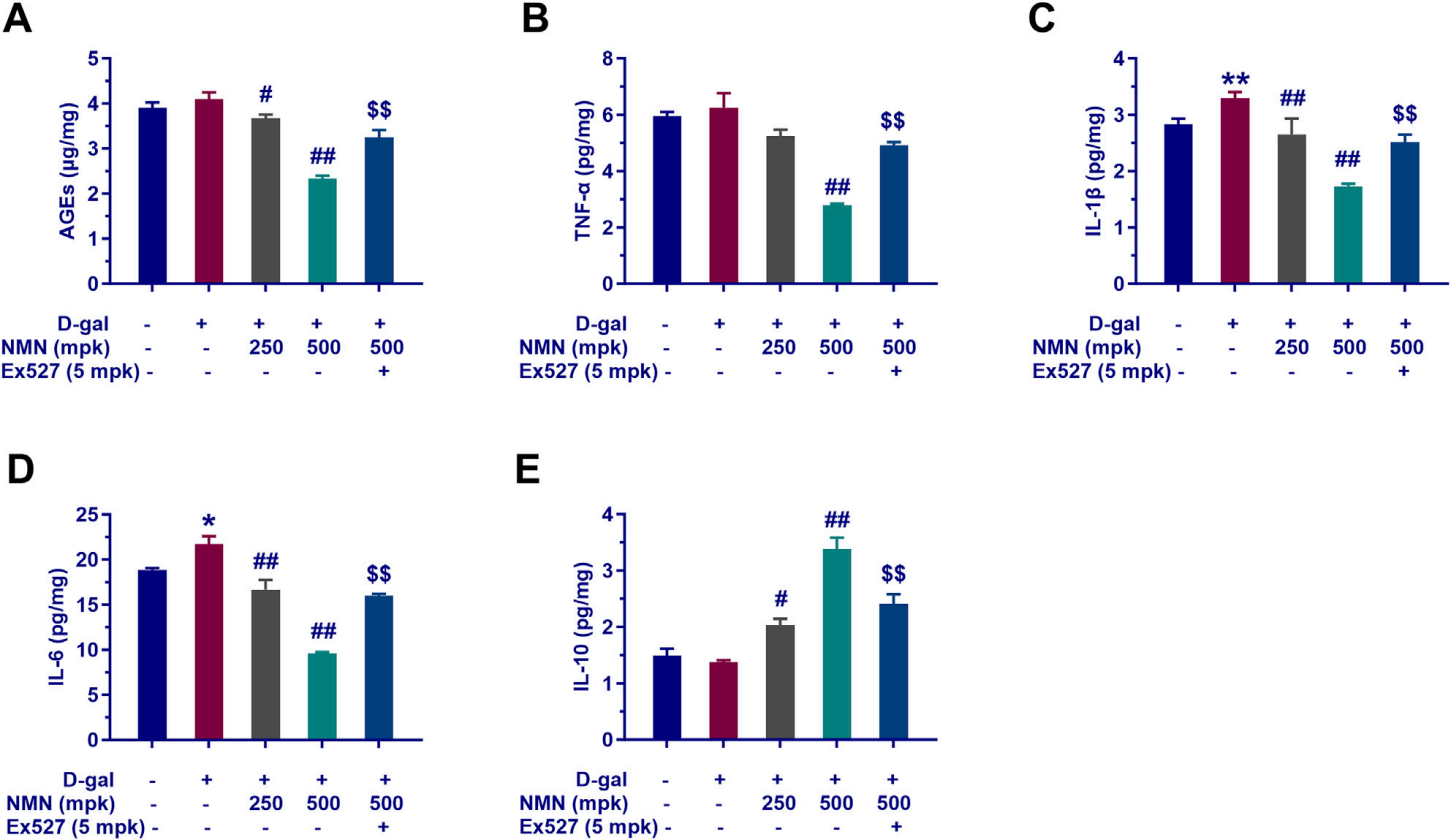
NMN exerts anti-inflammatory effects and modulates AGEs of aging mice. Panels A through E showcase the quantification of AGEs **(A)**, TNF-α **(B)**, IL-1β **(C)**, IL-6 **(D)**, and IL-10 **(E)** in the mice brains assayed with radioimmunoassay kit. Each data point represents the mean ± SEM, with n = 6; **, p < 0.01, compared to the Normal group; #, p < 0.05, ##, p < 0.01, versus the D-gal group; $$, p < 0.01, in comparison to the NMN 500 mpk group.

In the context of aging research, nicotinamide mononucleotide (NMN) has emerged as a promising compound with potential anti-inflammatory effects. Our study rigorously assessed the impact of NMN on the attenuation of inflammatory markers and AGEs in aging mice. Following behavioral assessments, the cerebral cortex and hippocampus, key regions associated with cognitive function, were isolated and analyzed. We quantified AGEs and key inflammatory cytokines, including TNF-α, IL-1β, IL-6, and IL-10, using radioimmunoassay. NMN administration demonstrated a dose-dependent reduction in the expression of inflammatory markers in aging mice, including AGEs, TNF-α, IL-1β, and IL-6. The 500 mg/kg dose exhibited a particularly significant decrease in these markers (p < 0.01, Figures 4A–D), suggesting that NMN effectively mitigates the inflammatory response associated with aging. Concurrently, NMN at both 250 mg/kg and 500 mg/kg significantly upregulated the expression of the anti-inflammatory cytokine IL-10 (p < 0.05 and p < 0.01, Figure 4E), further attenuating inflammatory symptoms. Notably, co-administration of Ex527, a selective Sirt1 inhibitor, significantly inhibited the reduction in brain inflammation and AGEs induced by NMN at the 500 mg/kg dose (p < 0.01). This indicates a potential interaction between NMN and the Sirt1 signaling pathway in modulating inflammation and AGEs in aging mice. These findings highlight the potential of NMN in ameliorating inflammatory symptoms and reducing AGEs, both of which are critical factors in the pathogenesis of various age-related diseases.

### Effects of NMN on neurotransmitters of aging mice

The impact of nicotinamide mononucleotide (NMN) on the neurotransmitter profile in aging mice was investigated, with a particular focus on the modulation of key neurochemicals implicated in various neurological conditions. Our findings provide insights into the neuromodulatory effects of NMN, specifically on the levels of norepinephrine (NE), serotonin (5-hydroxytryptamine, 5-HT), glutamic acid, γ-aminobutyric acid (GABA), and dopamine (DA) in the brain.

**FIGURE 5:**
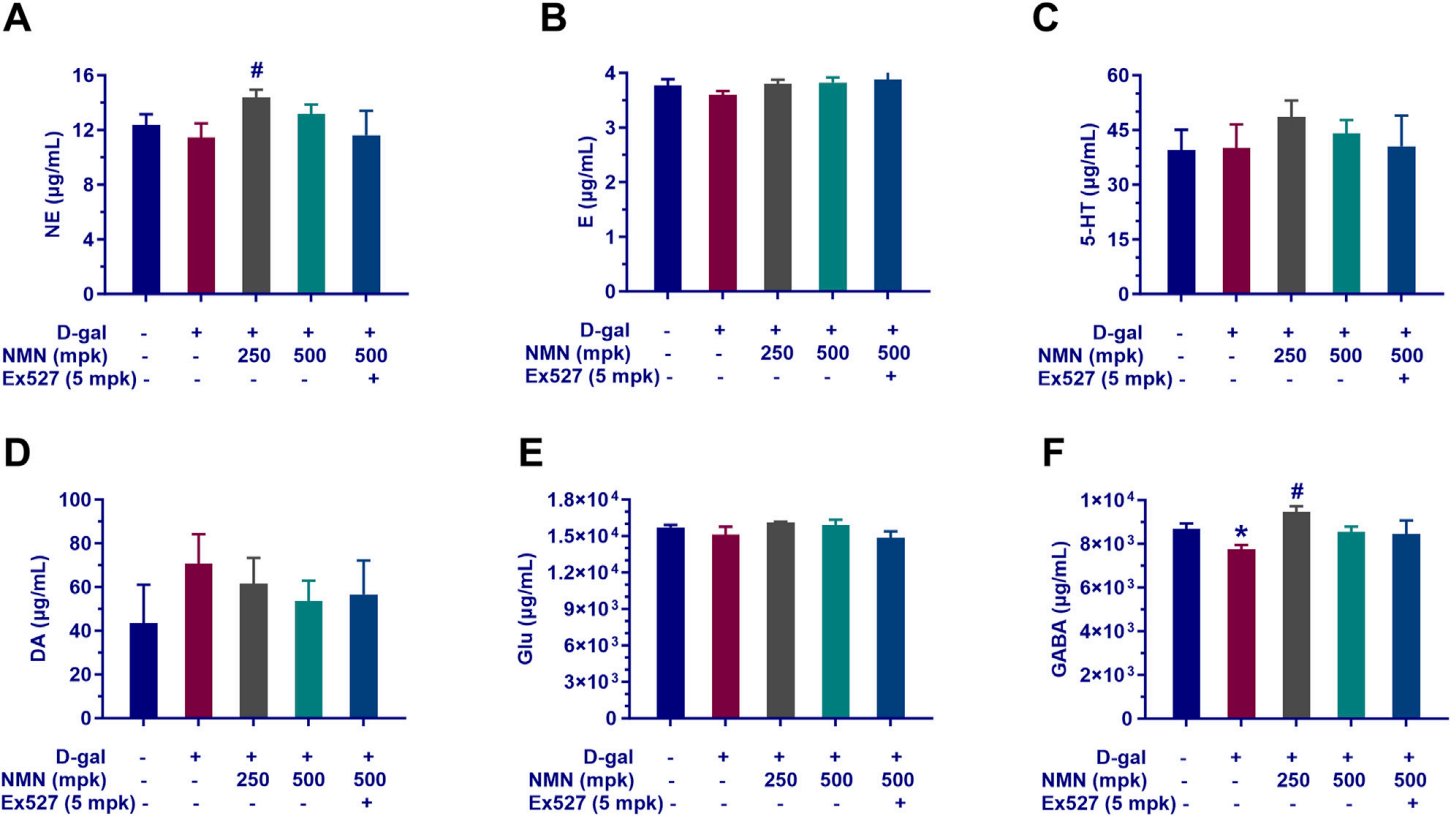
Influence of NMN on neurotransmitter levels in the brain tissue of aging mice. The key neurotransmitters in the brains of mice following the corresponding treatment regimens determined with LC-MS/MS assay indicated as **(A)**, Norepinephrine (NE); **(B)** Epinephrine; **(C)**, Serotonin (5-HT); **(D)**, Dopamine (DA); **(E)**, Glutamate (Glu); **(F)**, Gamma-Aminobutyric Acid (GABA). Each data point represents the mean ± SEM, with n = 5–6; *, p < 0.05, compared to the Normal group; #, p < 0.05, versus the D-gal group.

In the aging model, NMN administration at dosages of 250 and 500 mg/kg resulted in a dose-dependent increase in the levels of NE, serotonin, glutamic acid, and GABA (Figures 5A–E), suggesting a potential enhancement of the balance between monoaminergic and excitatory/inhibitory neurotransmitter systems. Notably, NMN at 250 mg/kg significantly elevated the levels of NE and GABA (p < 0.05; Figures 5A, E), both of which are critical in the regulation of arousal, mood, and inhibitory neuronal signaling. Conversely, NMN treatment led to a reduction in DA levels (Figure 5F), a neurotransmitter central to motor control, reward mechanisms, and motivation. Furthermore, the positive effects of NMN on neurotransmitter levels were partially reversed by co-administration of Ex527, a selective Sirt1 inhibitor, suggesting that the modulation of neurotransmitter dynamics by NMN may involve Sirt1-mediated pathways. This observation provides important insights into the potential mechanisms underlying NMN’s effects on cognitive and behavioral functions and underscores the role of Sirt1 in regulating neurochemical homeostasis in aging mice (p < 0.01). These findings contribute to our understanding of NMN’s role in modulating neurotransmitter systems and its potential therapeutic implications for neurological aging and related disorders.

### NMN regulates the aging-related genes in the brain of aging mice

**FIGURE 6:**
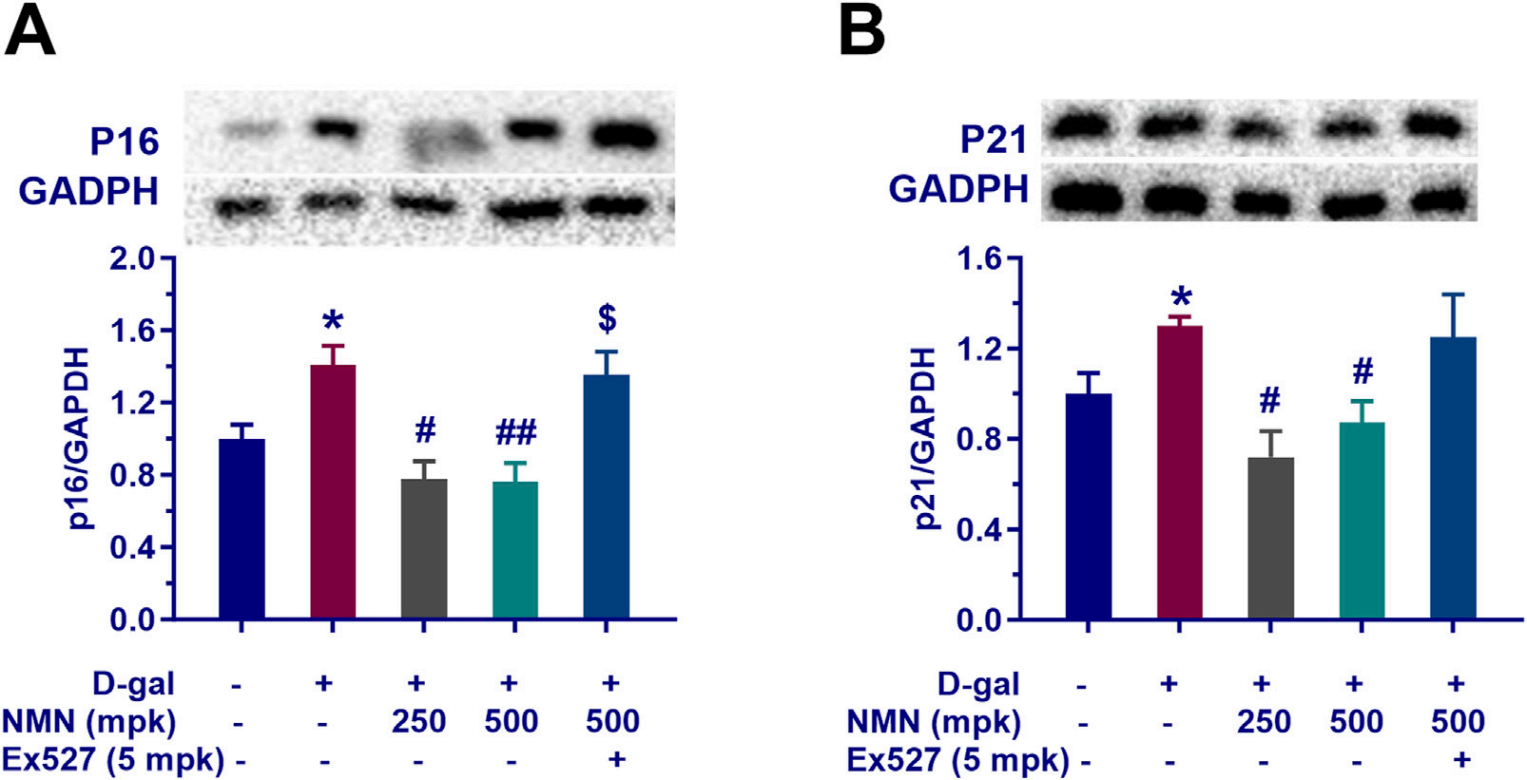
NMN mitigates the expression of senescence-associated proteins p16 and p21 of aging mice. Panels **(A, B)** indicate the protein levels of p16 and p21, respectively in the brains assayed by Western blotting with or without the treatment. Each data point represents the mean ± SEM, with n = 3; *, p < 0.05, compared to the Normal group; #, p < 0.05, ##, p < 0.01, versus the D-gal group; $, p < 0.05, in comparison to the NMN 500 mpk group.

In this study, we examined the regulatory effects of nicotinamide mononucleotide (NMN) on aging-associated genes in the brains of aging mice, with particular emphasis on p16 and p21, key regulators of cellular senescence. Our results demonstrated that NMN administration at dosages of 250 and 500 mg/kg significantly reduced the expression levels of p16 (p < 0.05; p < 0.01) and p21 (p < 0.05; p < 0.01) in brain tissue from aging mice (Figures 6A, B). This downregulation suggests a potential inhibitory effect of NMN on molecular markers of cellular aging, consistent with the well-established roles of p16 and p21 in mediating cell cycle arrest and promoting cellular senescence. Notably, co-administration of Ex527, a selective Sirt1 inhibitor, effectively counteracted the suppressive effects of NMN on p16 and p21 expression, leading to a significant increase in the levels of these proteins (p < 0.05). These findings are in agreement with prior studies implicating Sirt1 in the regulation of cellular senescence, as well as the expression of senescence-associated proteins such as p16 and p21. The modulation of these proteins by NMN may contribute to its observed anti-aging effects and provides a molecular framework through which NMN could exert its neuroprotective and anti-senescence activities in the context of brain aging. These results underscore the potential of NMN as a modulator of cellular aging pathways, offering novel insights into its therapeutic mechanisms for age-related neurodegenerative conditions and cognitive decline.

### Effect of NMN on mitochondria function in aging mice

**FIGURE 7:**
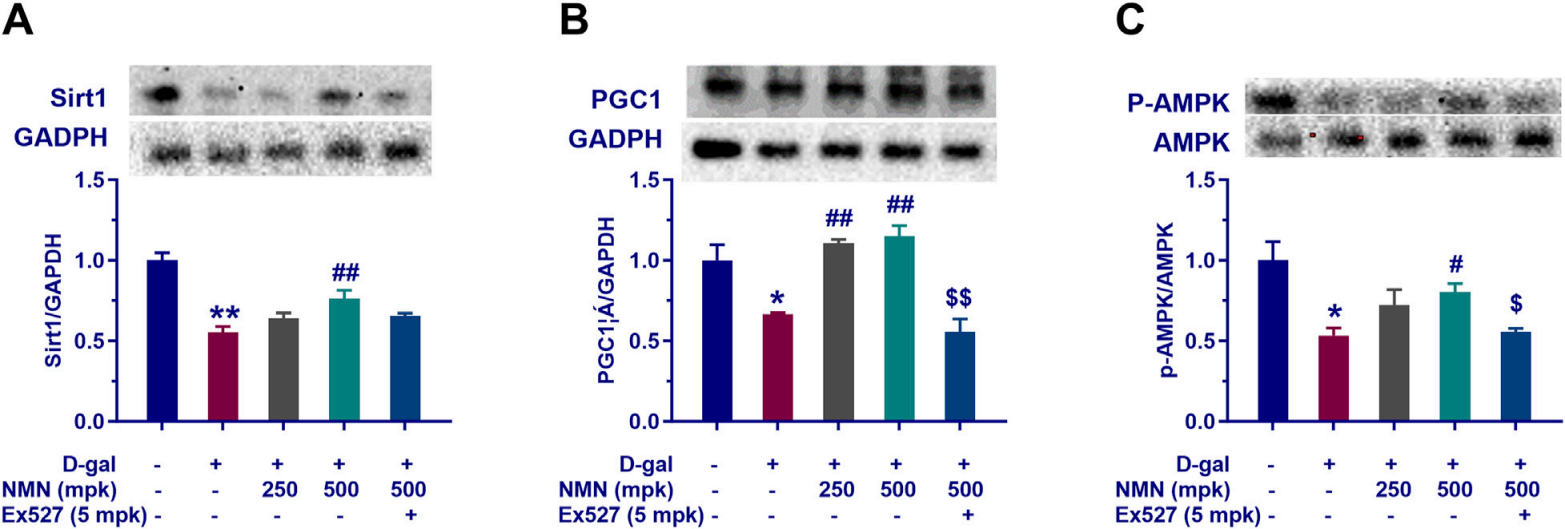
NMN reverses the decline of mitochondrial-related proteins tissue of aging mice. Panels **(A–C)** represent the protein expression levels of Sirt1, PGC1α, and phosphorylated AMPK (P-AMPK) in the brain assayed by Western blotting following the corresponding treatment, respectively. Each data point represents the mean ± SEM, with n = 3; *, p < 0.05, **, p < 0.01, compared to the Normal group; #, p < 0.05, ##, p < 0.01, versus the D-gal group; $, p < 0.05, $$, p < 0.01, in comparison to the NMN 500 mpk group.

In this study, we investigated the effects of nicotinamide mononucleotide (NMN) on mitochondrial function in aging mice, with particular focus on the Sirt1/AMPK/PGC-1α signaling pathway, a key regulator of mitochondrial biogenesis and energy metabolism. Our results demonstrated that NMN administration at dosages of 250 and 500 mg/kg significantly upregulated the expression of Sirt1, phosphorylated AMPK (p-AMPK), and PGC-1α in the brains of aging mice (Figures 7A–C; p < 0.05; p < 0.01). This upregulation suggests that NMN exerts a stimulatory effect on the Sirt1/AMPK/PGC-1α axis, which is central to the enhancement of mitochondrial biogenesis and cellular energy homeostasis. Furthermore, co-administration of Ex527, a selective Sirt1 inhibitor, abrogated the NMN-induced upregulation of the Sirt1/AMPK/PGC-1α pathway (p < 0.05; p < 0.01), indicating that the effects of NMN on mitochondrial function are, at least in part, mediated through Sirt1. These findings are consistent with previous studies that have highlighted the critical role of Sirt1 in regulating mitochondrial health and energy metabolism, particularly in the context of aging. In conclusion, our data suggest that NMN enhances mitochondrial function in aging mice through modulation of the Sirt1/AMPK/PGC-1α signaling pathway, providing molecular insights into its potential therapeutic effects on age-related mitochondrial dysfunction.

### NMN attenuates apoptosis of the brain of aging model mice

**FIGURE 8:**
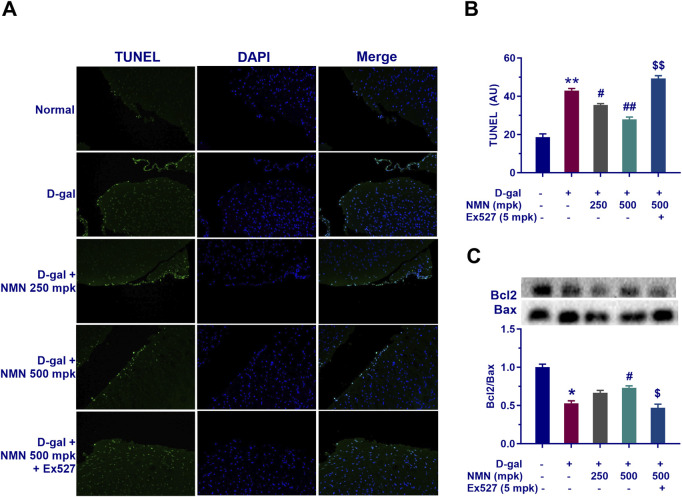
NMN mitigates apoptotic cell death in the brains of aging mice. **(A, B)** Representative images of TUNEL staining, a sensitive technique for detecting DNA fragmentation, a hallmark of apoptosis, along with corresponding statistical results in mice. **(C)** The protein levels of Bax and Bcl-2 as determined by Western blot analysis, along with their corresponding statistical results. Each data point represents the mean ± SEM, with n = 3; *, p < 0.05, **, p < 0.01, compared to the Normal group; #, p < 0.05, ##, p < 0.01, versus the D-gal group; $, p < 0.05, $$, p < 0.01, in comparison to the NMN 500 mpk group.

Subsequently, the regulatory effects and underlying mechanisms of NMN on D-gal-induced neuronal apoptosis in aging mice was performed. First, TUNEL staining demonstrated that D-gal treatment significantly increased the number of TUNEL-positive cells in brain tissues (p < 0.05), whereas NMN administration at doses of 250 and 500 mg/kg effectively reduced apoptotic cell proportions (Figures 8A, B), directly confirming its neuroprotective efficacy. Complementing these morphological findings, D-gal exposure markedly down-regulated the expression of the anti-apoptotic factor Bcl-2 while significantly elevating pro-apoptotic Bax levels (Figure 8C), resulting in a pro-apoptotic shift in the Bcl-2/Bax ratio (p < 0.05). Notably, NMN intervention not only reversed the decline in Bcl-2 expression but also suppressed the abnormal increase in Bax, thereby restoring the balance of mitochondrial apoptotic pathways and preserving cellular integrity. Mechanistically, co-administration of the selective Sirt1 inhibitor Ex527 substantially counteracted ameliorative effects of NMN on both the Bcl-2/Bax ratio and TUNEL-positive cell counts (p < 0.05; p < 0.01), suggesting the potential involvement of Sirt1 signaling activation in anti-apoptotic actions of NMN. Collectively, these results systematically illustrate the action of NMN in mitigating aging-associated neuronal apoptosis through modulating Bcl-2 family protein equilibrium and Sirt1-dependent pathways.

### NMN improves the structure of the damaged colon in aging mice

**FIGURE 9:**
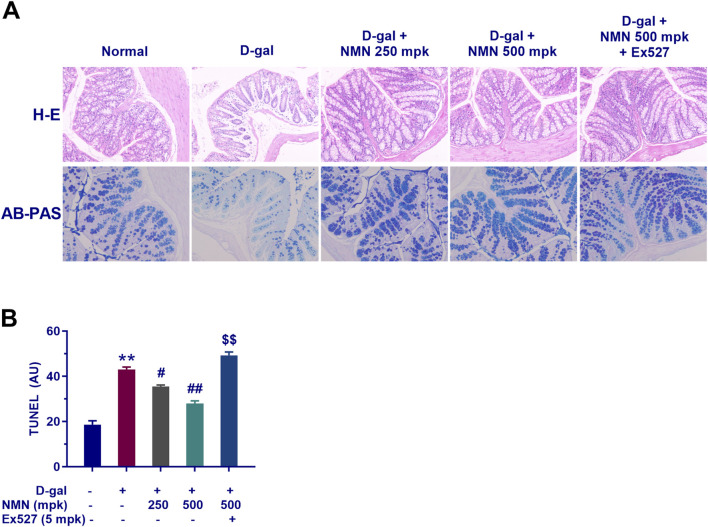
NMN exerts a protective effect on the intestinal mucosal barrier in aging mice. **(A)** Haematoxylin and eosin (HE) staining and Alcian Blue-Periodic Acid-Schiff (AB-PAS) staining of mouse colon sections, respectively. **(B)** The corresponding statistical results of AB-PAS staining. Each data point represents the mean ± SEM, with n = 3; **, p < 0.01, compared to the Normal group; #, p < 0.05, ##, p < 0.01, versus the D-gal group; $$, p < 0.01, in comparison to the NMN 500 mpk group.

Histological analysis of colonic tissues, using Hematoxylin and Eosin (H&E) staining, revealed distinct structural alterations in the model group treated with D-gal. The normal control group exhibited intact colonic wall architecture, characterized by well-organized mucosal microvilli, as well as normal crypt and gland structures. In contrast, the D-gal-treated group showed significant histopathological changes, including disorganized cellular arrangement in the colonic mucosa, partial crypt destruction, glandular atrophy, thinning of the submucosa, and pronounced eosinophilic infiltration, all of which are indicative of an inflammatory response and tissue damage. However, treatment with NMN at doses of 250 and 500 mg/kg preserved mucosal integrity, maintained the organization of goblet cells, and prevented epithelial cell shedding. Additionally, neutrophil infiltration was reduced, and there was a marked recovery in both crypt depth and villus height, suggesting a restorative effect of NMN on the colonic epithelial barrier (Figure 9A). Furthermore, Alcian Blue-Periodic Acid-Schiff (AB-PAS) staining demonstrated a dose-dependent increase in the number of goblet cells in the colons of NMN-treated mice. Goblet cells, essential for maintaining mucosal integrity and barrier function, were significantly enhanced (Figure 9A). Co-administration of Ex527, a selective Sirt1 inhibitor, abrogated the beneficial effects of NMN on D-gal-induced colonic degeneration, as evidenced by the reversal of these improvements (Figure 9B; p < 0.01). This suggests that the protective effects of NMN on colonic architecture may be partially mediated through the Sirt1 pathway, a key regulator of cellular health and longevity. These findings highlight the potential therapeutic utility of NMN in mitigating age-related intestinal dysfunction and inflammation.

## Discussion

NAD^+^ is a critical coenzyme involved in redox reactions and functions as a vital cofactor for non-redox enzymes, including sirtuins and poly (ADP-ribose) polymerases (PARPs), which are essential for maintaining tissue homeostasis and metabolic balance. A growing body of evidence underscores the causal relationship between cellular NAD^+^ levels and the aging processes of various tissues, such as the skin, blood, liver, muscle, and brain (Covarrubias et al., 2021). Aging is characterized by a progressive decline in NAD^+^ concentrations across multiple human tissues, which is considered a key contributor to the onset of age-related diseases. Disruptions in metabolic states associated with aging can result in the depletion of NAD^+^ levels, which, in turn, exacerbate the inflammatory response through the upregulation of pro-inflammatory cytokines. This elevation in cytokines triggers cellular and DNA damage, further activating NAD^+^-consuming enzymes such as CD38 and PARP, leading to an accelerated depletion of NAD^+^. In aged brain tissue, oxidative stress, mitochondrial dysfunction, and impaired autophagy contribute to increased DNA damage. Accumulated DNA damage activates PARP, further depleting NAD^+^ levels within the nucleus. The consequent reduction in NAD^+^ leads to diminished Sirt1 activity, which impairs mitochondrial function and exacerbates oxidative stress, creating a feedback loop of cellular damage. Furthermore, axonal degeneration, a precursor to numerous neurodegenerative diseases, is closely linked to a rapid decline in NAD^+^ levels, attributed to decreased expression of the NAD^+^ biosynthetic enzyme nicotinamide mononucleotide adenylyl transferase 2 (NMNAT2). Supplementation with nicotinamide mononucleotide (NMN) has been shown to increase NAD^+^ levels, activate the Sirt1 pathway, and enhance the expression of the mitotic checkpoint kinase BubR1, extending lifespan in murine models (North et al., 2014). Similarly, a one-week NMN intervention in aged mice improved mitochondrial oxidative metabolism, restored mitochondrial homeostasis, and reversed skeletal muscle dysfunction, bringing muscle health to levels comparable to those of young mice (Gomes et al., 2013). These findings highlight the potential of NMN as a therapeutic strategy for combating age-related declines in NAD^+^ and mitigating tissue dysfunction. In the current study, C57BL/6 mice were treated with D-gal for 8 weeks, inducing age-related phenotypic changes and confirming the successful establishment of an aging model. Administration of NMN over the same period ameliorated the senescence phenotype in D-gal-treated mice. Notably, body weight, a critical indicator of overall health, did not exhibit significant fluctuations in the NMN-treated groups, suggesting that long-term supplementation at a dose of 500 mg/kg/day was well-tolerated. However, the co-administration of NMN (500 mg/kg) and Ex527, a Sirt1 inhibitor, resulted in the slowest weight gain among the aging mice. This outcome is consistent with reports by Jonathan et al., who found that Ex527-induced Sirt1 inhibition increased p53 acetylation without affecting cell viability or gene expression in etoposide-treated cells (Solomon et al., 2006). Similarly, Kundu et al. demonstrated that Ex527 administration in high-fat diet-induced diabetic rats significantly reduced elevated blood glucose levels and kidney weight (Kundu et al., 2020). The precise effects of Ex527 on body weight remain to be elucidated. However, NMN supplementation notably improved neurological function and mitigated cognitive deficits induced by D-gal, with Sirt1-mediated pathways playing a pivotal role in the neuroprotective effects of NMN. However, we acknowledge that the AMPK-PGC-1α axis may crosstalk with other pathways (e.g., Nrf2/ARE, NF-κB). The potential synergistic involvement of other pathways cannot be excluded (Cheng et al., 2024; Salminen et al., 2011).

The elevation of NAD^+^ levels has become a primary focus in aging research, with a growing recognition of its potential to slow tissue aging and preserve functional integrity. NAD^+^ can be synthesized through three distinct pathways: the *de novo* synthesis pathway (also known as the kynurenine pathway), the Preiss-Handler pathway, and the salvage pathway. The *de novo* pathway, which occurs primarily in the liver, begins with dietary tryptophan. The Preiss-Handler pathway converts dietary niacin to NAD^+^, while the salvage pathway is the principal route for NAD^+^ biosynthesis in most mammalian cells. Through this pathway, nicotinamide (NAM) is converted into NMN by nicotinamide phosphoribosyltransferase (NAMPT), and NMN is subsequently converted into NAD^+^ by NMNAT. Therefore, direct supplementation with NMN is an effective strategy to enhance the salvage pathway (Song et al., 2023). NMN, a natural NAD^+^ intermediate, is widely found in foods such as vegetables, fruits, and meats, with particularly high concentrations in plant-based foods such as soybeans, avocados, broccoli, cabbage, and cucumbers. While physiological amounts of NMN can be absorbed through dietary sources, supplemental NMN is often necessary under pathological conditions such as aging to counteract the decline in intracellular NAD^+^ levels. The safety of NMN supplementation has been evaluated in both preclinical and clinical studies. In rodent models, administration of 500 mg/kg/day of NMN for 91 days did not result in adverse events, while a higher dose of 2000 mg/kg/day caused weight loss and reduced food intake (Turner et al., 2021). Long-term studies have shown that NMN supplementation in mice for 12 months is safe, inhibits age-related weight gain, improves energy metabolism, enhances physical activity, and increases insulin sensitivity (Mills et al., 2016). Similarly, nicotinamide riboside (NR), another NAD^+^ precursor, has been shown to delay neural stem cell aging and extend lifespan in mice (Zhang et al., 2016). A Phase I clinical trial found that oral NR supplementation was safe and resulted in minor clinical improvements in Parkinson’s disease patients (Brakedal et al., 2022). Furthermore, NR supplementation activates the mitochondrial unfolded protein response and enhances neurogenesis in amyotrophic lateral sclerosis (ALS) mouse models (Zhou et al., 2020). Beyond NAD^+^ precursors, NAMPT activators such as P7C3 and its analog P7C3-A20 have been explored for their ability to enhance NAD^+^ salvage pathway activity. These activators increase intracellular NAD^+^ levels and offer neuroprotective effects in various animal models of neurodegeneration and injury (Wang et al., 2014). Additionally, natural compounds such as triterpenes from Panax notoginseng and flavonoids like apigenin have demonstrated neuroprotective effects by modulating NAD^+^ levels (Xie et al., 2020; Escande et al., 2013). *Rehmannia glutinosa*, a traditional Chinese medicine, has also been shown to increase NAD^+^ levels and enhance energy metabolism, contributing to its neuroprotective properties (Han et al., 2022).

Cognitive and memory decline associated with aging was reversed by NMN supplementation, as evidenced by OFT and MWM performance, consistent with previous reports (Hosseini et al., 2019; Liu et al., 2022). These findings suggest that NMN may exert neuroprotective effects and enhance cognitive function in aging mice, likely through modulation of sirtuin pathways and NAD^+^ levels. Neurodegenerative diseases, including AD, Parkinson’s disease, and ALS, are characterized by oxidative stress, mitochondrial dysfunction, and neuroinflammation, all of which are exacerbated by aging. AGE formation, either through endogenous processes or dietary intake, is associated with cellular aging and plays a role in the pathogenesis of age-related diseases, contributing to oxidative stress and neuroinflammation. NMN has been shown to mitigate these effects by modulating oxidative stress markers (e.g., SOD, CAT) and inflammatory cytokines (e.g., TNF-α, IL-1β, IL-6), thus underscoring its potential in protecting against neurodegenerative diseases.

In addition to its effects on cognitive function, NMN supplementation ameliorates intestinal barrier dysfunction, a common age-related change, suggesting its broader impact on aging in both the gastrointestinal and central nervous systems (Huang et al., 2022). NMN enhances mitochondrial function by increasing NAD^+^ levels, promoting cellular energy production, and maintaining intestinal epithelial integrity. This mechanism may be particularly beneficial in conditions such as inflammatory bowel disease, where impaired intestinal barrier function exacerbates inflammation. NMN-mediated activation of sirtuins further attenuates inflammation by deacetylating histones and transcription factors, thereby downregulating pro-inflammatory genes (Kiss et al., 2020). Moreover, NMN influences microbial metabolism and gut microbiota diversity, which plays a crucial role in immune modulation and gastrointestinal homeostasis. NMN supplementation has been shown to alter gut microbial composition and metabolome, including shifts in the abundance of beneficial bacteria such as *Helicobacter*, Lachnospiraceae, and *Faecalibacterium*, which are associated with improved nicotinamide metabolism (Niu et al., 2021).

Furthermore, NMN supplementation corrected age-related declines in neurotransmitter levels, particularly norepinephrine (NE), which plays a key role in regulating both neural function and intestinal barrier integrity. NE, as the primary neurotransmitter of the sympathetic nervous system, is crucial for maintaining the intestinal barrier, while GABAergic signaling in the enteric system promotes intestinal defense and immunity (Deng et al., 2023; Lee et al., 2005; You et al., 2021). These findings highlight the intricate interplay between neurotransmitters, NAD^+^ metabolism, and intestinal health, suggesting that NMN may offer therapeutic benefits through these interconnected pathways.

The study demonstrates that NMN supplementation in D-gal-induced aging mice improves cognitive function, mitochondrial health, and intestinal barrier integrity, largely via Sirt1 activation and NAD^+^ restoration. Translating these findings to humans requires cautious optimism, considering both conserved biological pathways and interspecies differences. NAD^+^ metabolism and sirtuin pathways are evolutionarily conserved, suggesting similar benefits in humans. Clinical trials with NAD^+^ precursors like NR have shown safety and modest efficacy in neurodegenerative diseases (e.g., Parkinson’s) and metabolic disorders, supporting the potential of NMN (Brakedal et al., 2022). D-gal-induced aging mimics accelerated oxidative stress and inflammation but does not fully replicate natural aging. Human aging involves multifactorial processes (e.g., epigenetics, chronic diseases), which may alter NMN’s efficacy. Phase I trials with NR show promise, but NMN-specific human data are limited. Although the clinical trial reported NR’s safety in Parkinson’s patients, hinting at NMN’s potential. However, long-term studies are needed to confirm benefits on cognition and longevity. NMN is naturally present in foods (e.g., broccoli, avocado), suggesting low toxicity, but synthetic supplementation’s long-term effects remain unproven. Regulatory approval and standardization (e.g., purity, bioavailability) are critical for clinical translation. Human equivalents require careful scaling, as high doses in mice (2000 mg/kg) caused adverse effects, though lower doses in humans (e.g., 250–1,000 mg/day) have been tolerated in early trials (Yoshino et al., 2018).

Long-term NMN supplementation is more necessary for addressing age-related clinical symptoms. A 12-month NMN study in mice inhibited age-related weight gain, improved insulin sensitivity, and enhanced physical activity without adverse effects, indicating sustained benefits (Mills et al., 2016). Similarly, NR extended lifespan in mice by delaying (Zhang et al., 2016). These findings suggest that prolonged NMN use may amplify benefits, particularly in mitigating chronic age-related pathologies. High-dose NMN (2000 mg/kg) in rodents caused weight loss and reduced food intake, underscoring the need for dose optimization (Turner et al., 2021). Chronic use might theoretically downregulate endogenous NAD^+^ synthesis (e.g., NAMPT activity), though no evidence currently supports this. Aging in humans spans decades, necessitating decades-long studies to assess NMN’s impact on longevity and age-related diseases. Short-term human trials (≤12 weeks) show NAD^+^ elevation and metabolic improvements, but longer trials are needed to evaluate cognitive preservation, cancer risk, and organ-specific effects (Chen et al., 2024). NMN’s modulation of gut microbiota (e.g., increased Faecalibacterium) and intestinal barrier function may have systemic anti-aging effects (Huang et al., 2022). Long-term studies should explore whether these changes reduce inflammation-driven pathologies like Alzheimer’s or inflammatory bowel disease. Co-administration with NAMPT activators (e.g., P7C3) or lifestyle interventions (e.g., exercise, caloric restriction) could synergize with NMN to enhance NAD^+^ levels, warranting investigation in extended studies (Das et al., 2018; Khaidizar et al., 2021; Wang et al., 2021).

Despite the promising results, the current study has certain limitations. The exact mechanisms by which NMN crosses the blood-brain barrier and its direct effects on neuronal cells require further investigation. Additionally, long-term clinical studies are needed to explore the dose-response relationships, potential side effects, and individual variability in response to NMN supplementation. These studies will be critical in optimizing the clinical application of NMN for aging-related diseases.

In conclusion, NMN supplementation improves cognitive function and mitigates age-related intestinal barrier dysfunction in mice. These effects are likely mediated through the correction of oxidative stress, reduction of inflammation, and modulation of senescence and apoptosis-related pathways. The present study suggests that NMN holds significant therapeutic potential for the treatment of aging-associated diseases, including neurodegenerative diseases and impaired intestinal barrier function. However, the regulation of related signaling pathways by Sirt1 underscores the need for further exploration of these mechanisms to fully leverage the therapeutic potential of NMN.

## Data Availability

The raw data supporting the conclusions of this article will be made available by the authors, without undue reservation.
